# Portal Vein Embolization for Future Liver Remnant Enhancement and Combined Modality Treatment for the Management of Post-hepatic Resection Biliary Fistula in an 18-Month Old Child With Hepatoblastoma

**DOI:** 10.3389/fsurg.2019.00054

**Published:** 2019-09-13

**Authors:** Odaiyappan Kannappan, Keduovinuo Keditsu, Monica Bhagat, Anurag Shrimal, Ashwin Polnaya, Suyash Kulkarni, Sajid S. Qureshi

**Affiliations:** ^1^Division of Pediatric Surgical Oncology, Department of Surgical Oncology, Tata Memorial Centre, Mumbai, India; ^2^Department of Interventional Radiology, Tata Memorial Centre, Mumbai, India

**Keywords:** hepatoblastoma, hepatic resection, portal vein embolization, future liver remnant (FLR), post-operative biliary leak, percutaneous transhepatic biliary drainage (PTBD)

## Abstract

Hepatic resection is the mainstay of treatment for hepatoblastoma. However, the presence of adequate future liver remnant (FLR) is essential to prevent postoperative liver failure. Portal vein embolization (PVE) is commonly utilized in adults for promoting hypertrophy of FLR, however, it is sparingly used in children. Secondly, bile leak after liver resections is a well-defined complication. Apart from conservative treatment such as drainage and antibiotic, several management strategies including endoscopic, percutaneous, and surgical approaches have been described for its management. We present an 18-month old child with hepatoblastoma for whom PVE was performed to enhance the FLR so that an extended right hepatectomy could be accomplished. The same patient endured delayed postoperative biliary leak wherein the conservative, and non-operative interventional procedure failed, however, surgery combined with intraoperative interventional radiology procedure was utilized with a favorable outcome.

## Case Report

**Figure 1:**
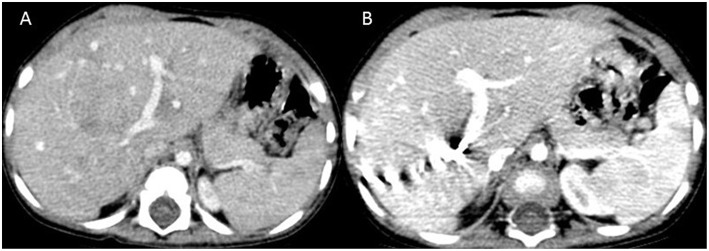
Computed tomography images at the level of splenic hilum before **(A)** and after **(B)** portal vein embolization showing hypertrophy of segment 3.

**Figure 2:**
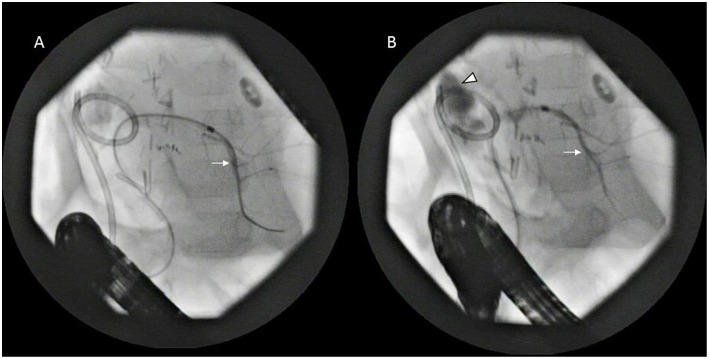
Endoscopic retrograde cholangiopancreatography (ERCP)—the left biliary tree (arrow) is cannulated **(A)** which is well-demonstrated with contrast **(B)** including the extravasation at the hilum (arrowhead). The percutaneously placed pigtail drain is also seen.

**Figure 3:**
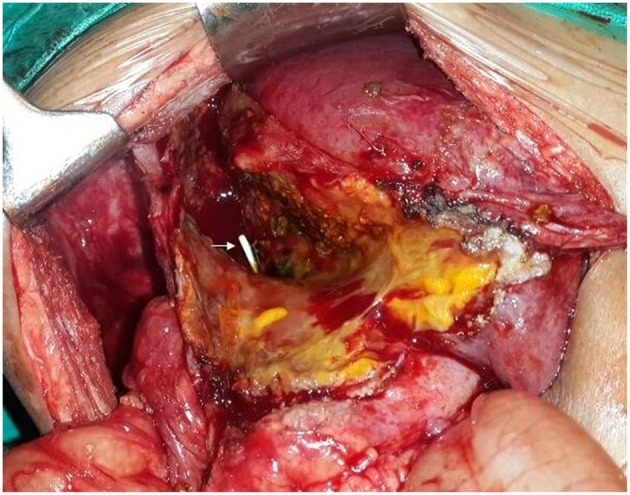
Intraoperative photograph showing the stent protruding into the peritoneal cavity (arrow) from the hepatic duct confluence.

**Figure 4:**
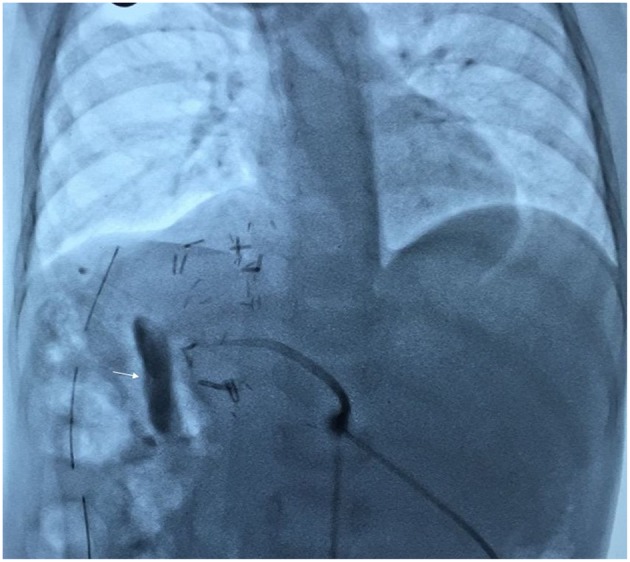
Fluoroscopy image showing completed hepaticojejunostomy with internoexternal stent with contrast in the jejunal limb (arrow).

An 18 months old male child presented with an accidentally detected abdominal mass. Radiological examination with a triphasic computerized tomography (CT) scan showed multifocal masses in segments IVB, V, VI, and VIII of the liver with associated an extrahepatic extension. The serum alpha-fetoprotein (AFP) was 7,109 ng/ml [normal: 0–9 ng/ml]. The staging assigned was PRETEXT IIIE. An image-guided biopsy was suggestive of fetal epithelial type hepatoblastoma. The patient received six cycles of superPLADO chemotherapy i.e., cisplatin, doxorubicin, and carboplatin. A response evaluation after completion of chemotherapy revealed a fall in AFP levels to 4,049 ng/ml and a marginal decrease in size on CT scan (POSTEXT III). Extended right hepatectomy was planned, however, the estimated future liver remnant (FLR) was 26.8% of the liver volume. In view of the borderline FLR, a preoperative portal vein embolization (PVE) was considered to reduce the chance of postoperative liver failure. A 4F catheter was used for PVE with polyvinyl alcohol (PVA) particle of the right branch and one more cycle of chemotherapy was offered as a bridge to surgery. After 25 days the FLR enlarged to 50% of the liver volume with a volume gain of 104% and kinetic growth rate of 5.8% per week (Figure 1) (1). An extended right hepatectomy was performed. The liver parenchyma was transected with a combination of Kelly clysis and harmonic scalpel. The right hepatic duct was secured further to the confluence of the bile duct and the integrity of the left duct was ensured. The postoperative recovery was uneventful and the child was discharged on the seventh postoperative day. The histopathology revealed residual mitotically active fetal type hepatoblastoma with minimal response to chemotherapy (99% viable tumor). The resection margins were more than 2 cm away and free of tumor cells. The child presented with fever, four weeks after the surgery. Ultrasonography (USG) revealed a cystic collection in the hepatorenal pouch for which a percutaneous drainage catheter was placed which drained bile. The biliary leak persisted with no regression in the daily drain output (>100 ml/day). A hepatobiliary iminodiacetic acid (HIDA) scan was undertaken to identify the site of the leak which revealed bile leak from the hilum. A percutaneous transhepatic biliary drainage (PTBD) was attempted for diversion of the bile from the hilum, however, it was unsuccessful. An endoscopic retrograde cholangiopancreatography (ERCP) and a plastic stent were placed in the left hepatic duct, however, the bile leak persisted (Figure 2). With the failure of conservative, endoscopic, and percutaneous procedures surgery was offered. On exploration, the stent was found protruding into the peritoneal cavity at the hepatic duct confluence (Figure 3). The hilar structures were meticulously dissected amidst the severe inflammation secondary to bile leak. The caliber of the hepatic duct was 1.5 mm and under magnification side to side Roux-en-Y hepaticojejunostomy with 7-0 polypropylene sutures was performed. Due to the fragile nature of tissues, a PTBD was attempted intraoperatively to reinforce the anastomosis. After successful cannulation of the biliary system, an interno-external stent was placed across the anastomosis (Figure 4). The bile leak stopped postoperatively and the child recovered well. The child completed the remaining three cycles of adjuvant chemotherapy and is disease free at 2 years after completion of treatment.

## Discussion

The present case highlights firstly, the successful use of PVE for promoting hypertrophy of FLR and secondly, the management of an incessant postoperative biliary fistula with a multimodal approach after the failure of non-operative measures. Adequate FLR is essential to prevent postoperative morbidity and liver failure following any major hepatic resections. Although there is no consensus on minimal FLR required in the pediatric population, the adult threshold of 30% in the non-cirrhotic liver is applied to children (2). The child described in this report had a borderline FLR value of 26.8% for which PVE of the right branch was performed. The FLR increased to 50% which is almost twice the FLR prior to PVE. PVE possibly causes the tumor to shrink and its border with healthy liver becomes clearly demarcated. It causes apoptotic necrosis of the embolized liver and triggers hypertrophy of the remnant healthy liver by means of growth factor production and redirection of portal flow toward the FLR (3). PVE has 95–100% technical and clinical success rates with <1% complications in adults (3). Only two anecdotal case reports of PVE or portal vein ligation in children have been described for liver tumors (4, 5). This deficiency is mostly due to the absence of tailored materials for children in interventional radiology compounded by the small caliber portal vessels which makes access to these vessels challenging (4). Nonetheless, the use of interventional radiology has markedly increased over the last three decades and have a favorable impact on therapy and outcomes in the treatment of pediatric solid tumors (6).

Although post hepatic resection bile leak rates have decreased to 5% from 10.8% in this decade compared to the past in adult's, they remain a major cause of postoperative morbidity, often leading to a prolonged hospital stay, delayed removal of abdominal drains, and need for additional (invasive) diagnostic tests and interventions (7). The biliary leaks rates after liver resection for hepatoblastoma in children are not well-described, however, institutional experience has reported as 12% (8). In our own experience of more than 100 liver resection for hepatoblastoma, the bile leak rate is 7% (unpublished). The conservative management for bile leak includes establishing secure drainage and antimicrobial treatment. Around 75% of the bile leaks resolve spontaneously with conservative management (9). Bile leak more than 100 ml/day after 10 days of diagnosis significantly predicts the necessity of interventional procedure (9). The commonly used non-operative interventional procedure includes ERCP sphincterotomy with stent placement or PTBD (7). Although, PTBD failed in the present case bile leak persisted even after ERCP and stent placement. Stents are placed with high success rates >90% and migration and perforation of biliary tract stents are rare complications (10). In the present case, although the stent was placed in the left hepatic duct across the confluence a probable migration and protrusion of the stent through the leakage site could be the reason for the persistent leakage.

Surgical exploration is indicated for the failure of conservative management and other interventional procedures or in the presence of diffuse peritonitis (9, 11). A Roux-en-Y hepaticojejunostomy was challenging in the present case in view of the adhesions at the porta due to surgery, bile leak and surrounding inflammation combined with the small caliber of the duct. Additionally, a PTBD with an interno-external stent was placed across the anastomosis. This approach was necessitated because conservative and interventional procedures have failed to control the bile leak earlier and another failure would be disastrous, therefore the need to reinforce the hepaticojejunostomy was considered. These procedures have been described independently for management of bile leak earlier, however, a combination of both have not been described. It remains debatable whether the integrity of the anastomosis or the presence of stent led to the healing of the leak, however, we believe both were complementary. The interno-external stent especially provided the assurance for bile drainage in the event of a failed hepaticojejunostomy.

## Conclusion

PVE is safe and beneficial for children to enhance the FLR for extended hepatic resections. The unrelenting bile leak post hepatic resection following the failure of conservative and non-operative intervention benefit with surgical exploration. A combination of the bilio-enteric anastomosis with intraoperative PTBD and an interno-external stent was useful in our patient for the healing of the biliary leak.

## Ethics Statement

Ethical review and approval was not required for the study on human participants in accordance with the local legislation and institutional requirements. Written informed consent to participate in this study was provided by the participants' legal guardian/next of kin. Written informed consent was obtained from the minor(s)' legal guardian/next of kin for the publication of any potentially identifiable images or data included in this article.

## Author Contributions

OK, SQ, and SK contributed conception and design of the study. OK and SQ organized the database. OK, KK, and MB wrote the first draft of the manuscript. AS, AP, SQ, and SK wrote sections of the manuscript. All authors contributed to manuscript revision, read, and approved the submitted version.

### Conflict of Interest Statement

The authors declare that the research was conducted in the absence of any commercial or financial relationships that could be construed as a potential conflict of interest.
